# Diagnostic Potential of Zinc Finger Protein-Specific Autoantibodies and Associated Linear B-Cell Epitopes in Colorectal Cancer

**DOI:** 10.1371/journal.pone.0123469

**Published:** 2015-04-13

**Authors:** Julie-Ann O’Reilly, Jenny Fitzgerald, Seán Fitzgerald, Dermot Kenny, Elaine W. Kay, Richard O’Kennedy, Gregor S. Kijanka

**Affiliations:** 1 Biomedical Diagnostics Institute, Dublin City University, Dublin, Ireland; 2 School of Biotechnology, Dublin City University, Dublin, Ireland; 3 Molecular and Cellular Therapeutics, Royal College of Surgeons in Ireland, Dublin, Ireland; 4 Department of Pathology, Royal College of Surgeons in Ireland and Beaumont Hospital, Dublin, Ireland; University of Pittsburgh, UNITED STATES

## Abstract

Colorectal cancer is one of the most common cancers worldwide with almost 700,000 deaths every year. Detection of colorectal cancer at an early stage significantly improves patient survival. Cancer-specific autoantibodies found in sera of cancer patients can be used for pre-symptomatic detection of the disease. In this study we assess the zinc finger proteins ZNF346, ZNF638, ZNF700 and ZNF768 as capture antigens for the detection of autoantibodies in colorectal cancer. Sera from 96 patients with colorectal cancer and 35 control patients with no evidence of cancer on colonoscopy were analysed for the presence of ZNF-specific autoantibodies using an indirect ELISA. Autoantibodies to individual ZNF proteins were detected in 10–20% of colorectal cancer patients and in 0–5.7% of controls. A panel of all four ZNF proteins resulted in an assay specificity of 91.4% and sensitivity of 41.7% for the detection of cancer patients in a cohort of non-cancer controls and colorectal cancer patients. Clinicopathological and survival analysis revealed that ZNF autoantibodies were independent of disease stage and did not correlate with disease outcome. Since ZNF autoantibodies were shared between patients and corresponding ZNF proteins showed similarities in their zinc finger motifs, we performed an *in silico* epitope sequence analysis. Zinc finger proteins ZNF700 and ZNF768 showed the highest sequence similarity with a bl2seq score of 262 (E-value 1E-81) and their classical C2H2 ZNF motifs were identified as potential epitopes contributing to their elevated immunogenic potential. Our findings show an enhanced and specific immunogenicity to zinc finger proteins, thereby providing a multiplexed autoantibody assay for minimally invasive detection of colorectal cancer.

## Introduction

Colorectal cancer is one of the most common cancers worldwide causing almost 700,000 deaths in men and women every year [1]. Detection of colorectal cancer at its early stage, when it is most likely to be curable, is vital and an impelling reason for the development of novel diagnostic assays suitable for population-wide screening. Current screening methods include faecal occult blood tests (FOBT), which are affected by poor patient uptake and low specificity and sensitivity, as well as the more invasive methods such as flexible sigmoidoscopy and colonoscopy, which are linked to potential complications and economic burden when employed as a primary screening tool in national programmes [2–4]. The detection of cancer-specific autoantibodies in the sera of patients offers an alternative route for minimally invasive population-wide cancer screening [5].

Cancer-specific autoantibodies are produced by the immune system in response to tumour-associated antigens (TAA). TAAs are self-proteins often overexpressed, mutated, misfolded or truncated in cancer cells throughout the process of tumourigenesis [6]. Detectable levels of autoantibodies specific to TAAs can be found at early stages of cancer and may act as natural signal amplifiers and excellent indicators of early disease [7]. Notably, the presence of autoantibodies may precede the development of clinical symptoms, providing an ideal rationale for pre-symptomatic autoantibody-based cancer screening [8–10]. In addition, multiplexing of tumour-associated antigens to panels of markers can potentially improve the specificity and sensitivity of diagnostic assays through the combined effect of individual autoantibodies profiles [11–13].

In this study, we aim to assess the utility of four zinc finger proteins as capture antigens for detection of autoantibodies in sera of patients with colorectal cancer. Zinc finger proteins (ZNF) are structurally defined by their evolutionarily conserved zinc finger motifs, which have been recognised as potential B-cell epitopes eliciting the production of autoantibodies in cancer and autoimmune disease [14–16]. We have previously identified autoantibodies to ZNF346, ZNF638, ZNF700 and ZNF768 in colorectal cancer patients using a 37,830-clone recombinant human protein array [17]. The four zinc finger proteins were overexpressed at the mRNA level in at least 20% of investigated tumours when compared to adjacent normal colorectal mucosa, thereby reflecting typical autoantibody incidence rates in cancer patients of 15–26% [12, 17]. The current study uses a clinically well-defined colorectal cancer cohort to develop a multiplexed ELISA-based autoantibody assay. We evaluate the specificity and sensitivity of individual autoantibodies and their combinations to detect cancer and we examine the relationship between autoantibody presence, clinical outcome and patient survival in a colorectal cancer cohort. In addition, we perform comparative sequence analyses to investigate potential cancer-specific autoimmune epitopes shared between the ZNF proteins.

## Materials and Methods

### Patients and samples

This study was approved by the Ethics (Medical) Research Committee at Beaumont Hospital, Dublin. Written informed consent was obtained from all patients. Patients undergoing colonoscopy were screened prospectively. The clinical notes of patients attending the colonoscopy clinic were reviewed and patients with a history of cancer, systemic inflammatory disease or autoimmune disease and patients taking immunosuppressive medication were excluded from the study. Colonoscopy findings were reviewed with the consultant physician and if a diagnosis of cancer or normal colonoscopy was made, then patients were eligible to participate. Subjects were then asked to provide a blood sample. Diagnosis of colorectal cancer was independently verified by a consultant pathologist. In total, sera from 96 patients with newly diagnosed colorectal cancer (CRC) and 35 patients with normal colonoscopies (non-cancer controls, NCC) were analysed in this study. All cases were diagnosed between 2001 and 2007 and a minimum of 5 years follow-up was recorded. Clinical characteristics of CRC and NCC subjects are shown in Table 1. Blood was obtained from all patients prior to chemo or radiation therapy and surgical treatment. Serum was prepared and stored at -80**°**C.

**Table 1:** Clinicopathological details of patient cohort.

	Colorectal cancer (CRC) (n = 96)	Non-cancer controls (NCC) (n = 35)
**Gender**		
Female	42	17
Male	54	18
**Age**		
Mean (SD*)	66 (12)	47 (15)
**History of polyps**	15	0
**Tumour site**		
Ascending colon	19	
Descending colon	4	
Traverse colon	19	
Sigmoid	16	
Rectal	35	
Not stated	3	
**Dukes stage**		
A	21	
B	31	
C	32	
D	11	
Not stated	1	
**Follow up (months)**		
Mean (SD*)	48 (20)	

* Standard Deviation

### Protein expression and purification

*E*. *coli* expression clones encoding recombinant human zinc finger genes ZNF346, ZNF638, ZNF700 and ZNF768 were obtained from imaGenes GmbH (Berlin, Germany) and were verified by sequencing (SourceBioscience Sequencing, Dublin, Ireland). ZNF proteins were overexpressed in *E*. *coli* and purified using nickel-NTA affinity chromatography. Briefly, auto-induction media (Tryptone 1% (w/v), Yeast 0.5%, 1mM MgSO_4_, 1x 5052, 1x NPS, 100 μg ml^-1^ carbenicillin and 50 μg ml^-1^ kanamycin) were inoculated with *E*. *coli* expression clones and incubated in a shaker incubator overnight (37°C, 200rpm). Bacteria were harvested after 24 hours by centrifugation (12,000g, 30 min), pellets were snap frozen in liquid nitrogen and stored at -20°C. Proteins were purified under denaturing conditions with 6M guanidine-HCl using nickel-NTA resin (Qiagen) and were size-verified using SDS-PAGE and Instant Blue staining. Protein concentrations were determined using a Nanodrop spectrophotometer (ND-1000, Thermo Scientific) at an absorbance of 280nm. Purified proteins were stored at -20°C.

### Autoantibody detection

An indirect ELISA was developed to detect autoantibodies in human serum. In brief, microtitre plates were coated with recombinant ZNF346, ZNF638, ZNF700 and ZNF768, respectively (overnight incubation (o/n) at 4°C; 100μL/well; 5 μg ml^-1^). Plates were washed, blocked (1 hr, 37°C), and incubated with human sera (o/n at RT; 100μL/well; 1 in 200 dilution). Following subsequent washing, plates were incubated with mouse anti-human IgG (1h at 37°C; 100μL/well; 1 in 10,000 dilution) and then with a peroxidase-labelled rabbit anti-mouse IgG (1h at 37°C; 100μL/well; 1 in 5,000 dilution). Tetramethylbenzidine was used as substrate for the peroxidase reaction (RT; 100μL/well) and the enzymatic reaction was stopped after 10 minutes with 1N HCl (RT; 50μL/well). The optical density (OD) was read at 450nm using an ELISA plate reader (Safire^2^, Tecan). Human IgG (50 μg ml^-1^) was used in duplicate as a control across microtitre plates. Optical density for the human IgG control was assigned a value of 1 OD. unit and used to determine relative OD. ELISA values.

### Epitope prediction analysis

Full-length amino-acid sequences for ZNF346, ZNF638, ZNF700 and ZNF768 were obtained from the PubMed website (http://www.ncbi.nlm.nih.gov/protein/) by searching sequencing data. The following NCBI references were identified: NP_036411.1 (ZNF346, 294 amino acids (aa)), NP_055312.2 (ZNF638, 1978aa), NP_653167.1 (ZNF700, 742aa) and NP_078947.3 (ZNF768, 540aa). Protein sequence similarities were analysed using BLAST. Non-intersecting protein sequence alignments were analysed using the local similarity program SIM (http://expasy.org/tools/sim-prot.html) adjusted for the PAM40 comparison matrix. Amino acid sequence pairs with ≥50% identity and between ≥5 to ≤20 amino acid sequence length were defined as similar, thereby representing typical length and amino acid complementarity of linear B-cell epitopes [18, 19]. Linear B-cell epitope prediction was conducted using BepiPred modified hidden Markov model with a threshold of 0.9 (0.25 sensitivity, 0.91 specificity) from Immune Epitope Database Analysis Resource v2.12 (http://tools.immuneepitope.org) [20].

### Statistical analysis

ELISA cut-offs were calculated as the average of the normal (NCC) relative OD values +2SD. Serum samples with ELISA values above the cut-off were identified as positive for autoantibodies against the protein of interest. Specificities and sensitivities for individual and grouped antigens were calculated according to Altman and Bland [21]. Briefly, specificity was calculated as the proportion of true negatives correctly identified as non-cancer controls by the assay (absence of ZNF autoantibodies) from the total number of non-cancer controls. Sensitivity was calculated as the proportion of true positives correctly identified as colorectal cancer patients by the assay (presence of at least one ZNF autoantibody) from the total number of colorectal cancer patients. Cumulative specificity and sensitivity was calculated by merging individual specificity and sensitivity values, whereby samples with more than one of the four ZNF autoantibodies contributed only once to the calculation. The correlation between the presence of autoantibodies and disease stage was assessed using Spearman rank correlation analysis. 5-year survival analysis was plotted according to the Kaplan-Meier method and the generalized log rank test was applied to compare the survival curves for each of the zinc finger proteins alone and in combination. Patients with follow-up information over 5-years were censored at year five post-diagnosis. All tests were analysed using GraphPad Prism 5 software (GraphPad Software, La Jolla, CA, USA) and the findings were considered statistically significant at *P <*0.05.

## Results

### ZNF-specific autoantibodies in CRC patients

**Fig 1:**
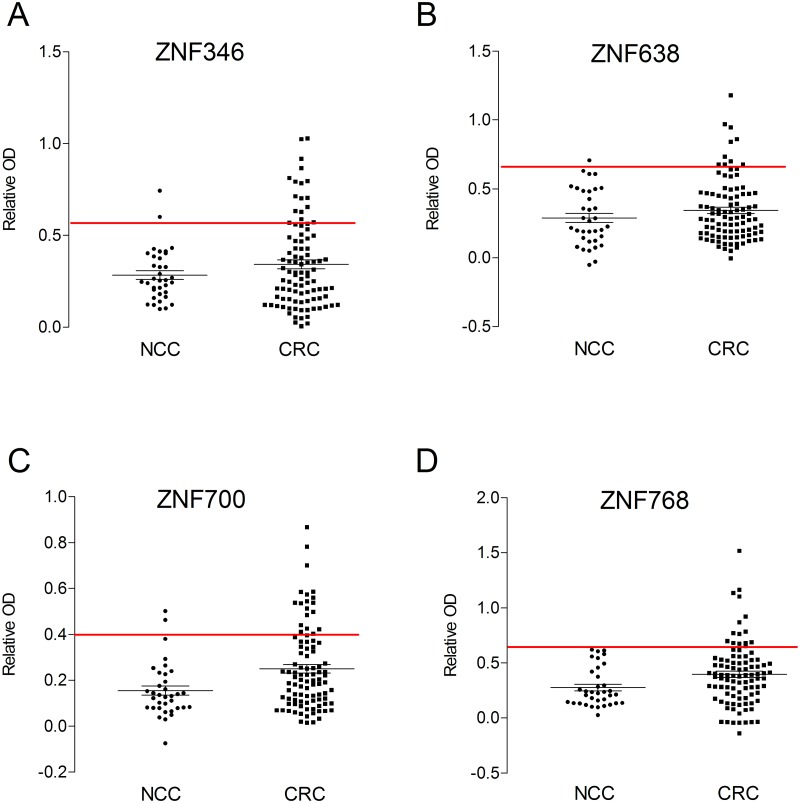
Relative OD autoantibody signals detected in CRC and NCC sera by indirect ELISA. Zinc finger proteins (A) ZNF346, (B) ZNF638, (C) ZNF700 and (D) ZNF768 were used as capture antigens. Black line represents the average of relative ODs (optical density) within each patient cohort. Red line represents ELISA cut-offs calculated as the average of NCC relative OD values +2SD. CRC: colorectal cancer, NCC: non-cancer control.

A total of 131 participants; 96 with a diagnosis of colorectal cancer (CRC) and 35 normal on colonoscopy (non-cancer controls, NCC) were enrolled in this study. Participant demographics and clinicopathological data are shown in Table 1. A serum-based ELISA for ZNF346, ZNF638, ZNF700 and ZNF768 was developed and autoantibodies to individual antigens were detected in 10–20% of CRC patients and in 0–5.7% NCCs. Autoantibodies to ZNF346 were detected in the sera of 15 out of 96 CRC patients (15.6%) and in 2 out of 35 non-cancer control samples (5.7%) (Fig 1A). Autoantibodies to ZNF638 were detected in sera of 10 out of 96 CRC patients (10.4%) and 1 out of 35 non-cancer control samples (2.9%) (Fig 1B). Autoantibodies to ZNF700 were detected in sera of 19 out of 96 CRC patients (19.8%) and in 2 out of 35 non-cancer control samples (5.7%) (Fig 1C). Autoantibodies to ZNF768 were detected in sera of 15 out of 96 CRC patients (15.6%) and were absent in non-cancer controls (0/35) (Fig 1D).

### Autoantibody panel enhances assay sensitivity

Next, we investigated whether multiplexing of autoantibodies to a multi-marker panel would improve specificity and sensitivity of the assay to correctly identify cancer patients from the CRC and NCC cohort. While individual sensitivities did not exceed 20% and specificities of individual ZNFs ranged between 94.3% and 100%, we observed a combination effect for the panel of markers (Table 2). The sensitivity of ZNF346 alone was 15.6% but when combined with ZNF638 it increased to 25%. Combination of ZNF346, ZNF638 and ZNF700 increased the sensitivity of the assay to 33.3%. Cumulative sensitivity of the overall assay using a combination of four zinc finger proteins as capture antigens was 41.7%, considerably greater than those of individual ZNF assays. With the two-fold increase in sensitivity, however, we have also observed a decrease in specificity in a four-marker panel (Table 2). While ZNF768 in our cohort was exclusively specific (100%) for CRC; the addition of ZNF346 resulted in a reduction in specificity to 94.3% and extending the panel further with ZNF638 and ZNF700 resulted in a cumulative specificity of 91.4% (Table 2).

**Table 2:** Frequency of autoantibodies to zinc finger proteins in patients.

Protein	Individual Specificity*	Individual Sensitivity**	Cumulative Specificity*	Cumulative Sensitivity**
ZNF346	94.3% (2/35)	15.6% (15/96)	94.3% (2/35)	15.6% (15/96)
ZNF638	97.1% (1/35)	10.4% (10/96)	91.4% (3/35)	25% (24/96)
ZNF700	94.3% (2/35)	19.8% (19/96)	91.4% (3/35)	33.3% (32/96)
ZNF768	100% (0/35)	15.6% (15/96)	91.4% (3/35)	41.7% (40/96)

* Positive for autoantibodies in non-cancer controls (NCC, n = 35)

** Positive for autoantibodies in colorectal cancer patients (CRC, n = 96)

### ZNF autoantibodies are independent of disease stage

Colorectal cancers were classified according to the Dukes’ staging system. 21 CRC patients were staged with Dukes A, 31 with Dukes B, 32 Dukes C and 11 Dukes D. ZNF autoantibodies were found to be distributed evenly across all disease stages (Table 3). There were no significant correlations between Dukes stages, early or late, and individual ZNF autoantibodies in sera of CRC patients. In addition, cumulative data for the four ZNF proteins showed no correlation with Dukes stage (Table 3). The absence of correlation between ZNF autoantibody presence and disease stage indicates a potential diagnostic relevance for both, early and advanced disease.

**Table 3:** Disease stage in CRC patients positive for ZNF-specific autoantibodies.

Dukes stage (n = 95)*	ZNF346 (n = 15)	ZNF638 (n = 10)	ZNF700 (n = 19)	ZNF768 (n = 15)	Cumulative
A (n = 21)	19% (4/21)	0% (0/21)	28.6% (6/21)	14.3% (3/21)	42.9% (9/21)
B (n = 31)	6.5% (2/31)	9.7% (3/31)	16.1% (5/31)	9.7% (3/31)	32.3% (10/31)
C (n = 32)	21.9% (7/32)	15.6% (5/32)	21.9% (7/32)	18.8% (6/32)	50% (16/32)
D (n = 11)	18.2% (2/11)	18.2% (2/11)	9.1% (1/11)	27.3% (3/11)	36.4 (4/11)

*Dukes stage in one patient not stated.

### ZNF autoantibodies are not prognostic of disease outcome

**Fig 2:**
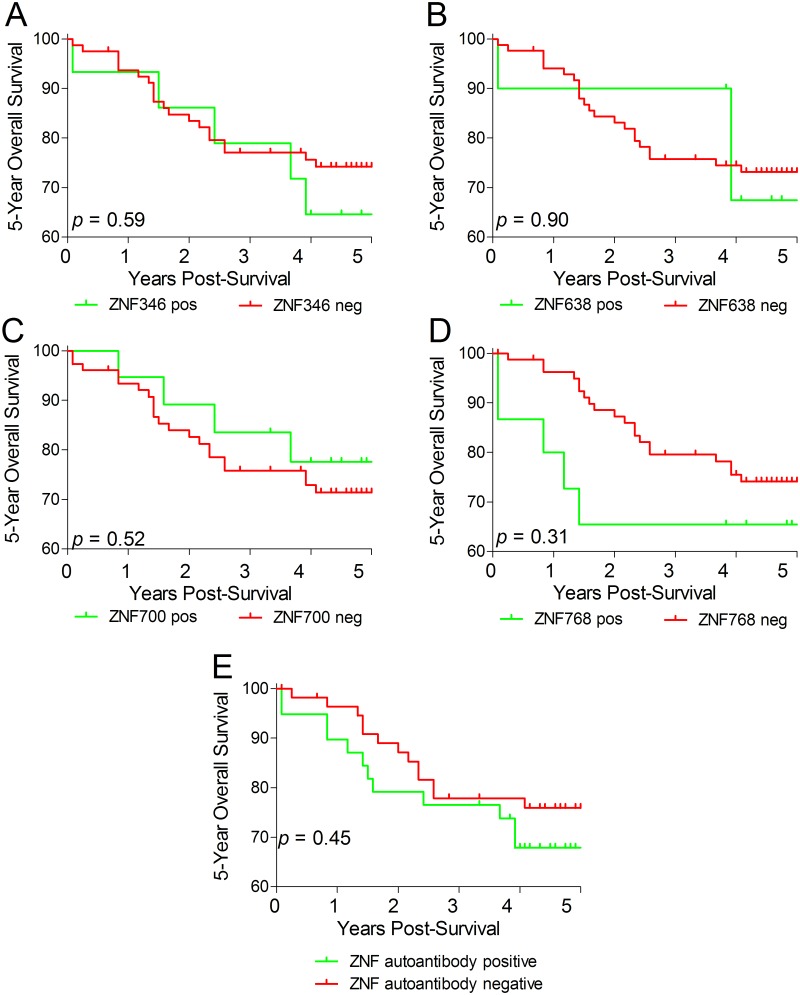
ZNF-specific autoantibodies and colorectal cancer prognosis. Kaplan-Meier analysis revealed that presence of autoantibodies specific to zinc finger proteins (A) ZNF346, (B) ZNF638, (C) ZNF700, (D) ZNF768 and (E) panel of all four ZNFs combined does not predict survival in colorectal cancer.

Kaplan-Meier analysis was performed to assess the association between patient survival and autoantibody presence (Fig 2). The 5-year overall survival (OS) analysis showed no correlation between patient outcome and presence of ZNF autoantibodies in sera of CRC patients. Furthermore, no prognostic value was seen when the four ZNF proteins were combined (Fig 2E).

### Classical C2H2 ZNF motif as immunogenic determinant

**Fig 3:**
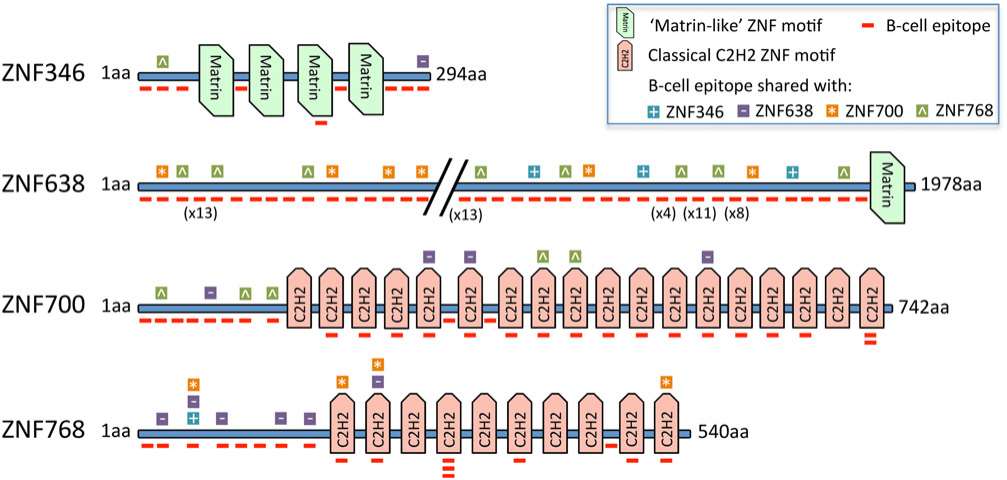
Epitope prediction analysis reveals the classical C2H2 ZNF motif as immunogenic determinant. Linear B-cell epitopes were predicted within zinc finger proteins ZNF346, ZNF638, ZNF700 and ZNF768 using the BepiPred method. Several B-cell epitopes were shared between all four zinc finger proteins. One B-cell epitope was located within the ‘matrin-like’ zinc finger motif of ZNF346. Seventeen B-cell epitopes were located within the classical C2H2 zinc finger motifs of ZNF700 and 8 were located within ZNF768. Five of the ZNF700 C2H2 B-cell epitopes and 4 of the ZNF768 C2H2 B-cell epitopes were also shared with other zinc finger proteins.

Autoantibody analysis in this study identified subsets of patients sharing serum autoantibodies to two or more ZNF proteins, which is in agreement with previous findings [17]. We have therefore set out to investigate whether sequence similarities in the ZNF motif regions may represent potential linear B-cell epitopes for ZNF-specific autoantibodies. The zinc finger proteins investigated in this study were classified as proteins containing either a ‘matrin-like’ zinc finger motif (ZNF346 and ZNF638) or a classical C2H2 zinc finger motif (ZNF700 and ZNF768) (Fig 3). Zinc finger proteins ZNF346 and ZNF638 share the ‘matrin-like’ motif with 4 repeats in ZNF346 and a single ‘matrin-like’ motif in ZNF638, whereas zinc finger proteins ZNF700 and ZNF768 share the classical C2H2 zinc finger motif with 10 and 18 repeats, respectively. The ZNF motif sequences are shown as Supporting Information in S1 Table.

As shown in Table 4, a two-sequence BLAST alignment (bl2seq) revealed the highest protein sequence similarity between ZNF700 and ZNF768 with a bl2seq score of 262 (E-value 1E-81), reflecting the numerous repeats of the classical C2H2 zinc finger motif in both proteins. To investigate peptide sequences representing potential shared epitopes we compared full-length sequences of all four zinc finger proteins using local similarity analysis SIM (Supporting Information, S2 Table). SIM analysis revealed 14, 19 and 21 sequence alignment pairs between ZNF638/ZNF700, ZNF638/ZNF768 and ZNF700/ZNF768, respectively (Table 4). Of these, only the ZNF700/ZNF768 alignments were frequently found within ZNF motif regions, with 9 alignments within ZNF700 zinc finger motifs and 10 alignments within ZNF768 zinc finger motifs (Table 4).

**Table 4:** Zinc finger protein sequence overlap and similarities.

ZNF protein pairs	bl2seq score	E value	SIM alignments*	SIM alignments within ZNF motifs	Shared B-Cell epitopes within SIM alignments	Shared B-Cell epitopes within ZNF motifs
ZNF346/ZNF638	21.6	0.17	6	2/0	1/3	0/0
ZNF346/ZNF700	21.2	0.073	12**	12**/11	0/0	0/0
ZNF346/ZNF768	20.8	0.063	3	2/2	1/1	0/1
ZNF638/ZNF700	19.2	1.9	14	0/3	7/4	0/3
ZNF638/ZNF768	23.9	0.058	19	0/3	8/6	0/1
ZNF700/ZNF768	261	1.00E-81	21	9/10	4/5	2/3

*Selection criteria: Identity >50%, exact match ≥5 ≤20 amino acids

**One ZNF346 epitope repeated 12 times in ZNF700

In order to investigate whether the identified SIM sequences may encode linear B-cell epitopes and potentially binding sites for serum autoantibodies, we performed epitope prediction analyses using BepiPred (Supporting Information, S3 Table) [20]. As shown in Table 5, linear B-cell epitopes were identified in all four zinc finger proteins with 9 epitopes predicted for ZNF346, 82 for ZNF638, 27 for ZNF700 and 17 for ZNF768. Of note, the amount of predicted epitopes closely relates to the length of the ZNF protein sequences. Numerous linear B-cell epitopes, however, were predicted within the classical C2H2 zinc finger motifs of zinc finger proteins ZNF700 and ZNF768, with 17 and 8 epitopes, respectively (Table 5). Only one B-cell epitope was found within the ‘matrin-like’ zinc finger motif of ZNF346 and none were found in ZNF638. When SIM alignments and B-cell epitope prediction data were combined, we found that all four zinc finger proteins contained multiple B-cell epitopes shared among the sequences (Fig 3). B-cell epitopes located within zinc finger motifs and also with high sequence similarity to one of the other ZNF protein sequences were identified only for the zinc finger proteins ZNF700 and ZNF768 (Fig 3), suggesting that the classical C2H2 zinc finger motif may account for an elevated immunogenic potential of these proteins.

**Table 5:** Predicted linear B-cell epitopes in ZNF proteins.

ZNF protein	B-cell epitopes	B-cell epitopes within ZNF motifs	B-cell epitopes within SIM alignments	B-cell epitopes shared by SIM and ZNF
ZNF346	9	1	2	0
ZNF638	82	0	18	0
ZNF700	27	17	9	5
ZNF768	17	8	8	3

## Discussion

Our results indicate that zinc finger proteins ZNF346, ZNF638, ZNF700 and ZNF768 are suitable for use as capture antigens in a blood-based biomarker assay for colorectal cancer. A significant increase in sensitivity and specificity of the assay was achieved when a panel of all four antigens was applied in a multiplex fashion. This data suggests that ZNF-based autoantibody assays may be suitable for patient screening, accompanying many of the enrolled national FOBT-based screening programs. However, the performance of the ZNF assay needs to be investigated in a larger, multi-site patient cohort, in other cancers and the panel may need to be extended to other known autoantibody markers. Interestingly, although the presence of ZNF autoantibodies did not correlate with disease outcome in this study, we observed that ZNF autoantibodies were independent of disease stage, suggesting their use for detection of non-symptomatic colorectal cancer patients, both in early and advanced stages of the disease. Furthermore, epitope analyses in this study predicted the classical C2H2 ZNF motif as a potential immunogenic determinant, suggesting a link between shared autoantibodies in CRC patients and autoantibody specificity to evolutionary conserved zinc finger structures in ZNF700 and ZNF768.

Our previous work identified the zinc finger proteins ZNF346, ZNF638, ZNF700 and ZNF768 as autoantigens from over 200 zinc finger proteins and several thousand other recombinant human proteins deposited on high-content protein arrays [17]. In the current study, we validated the presence of autoantibodies to ZNF346, ZNF638, ZNF700 and ZNF768 in colorectal cancer in an independent and larger patient cohort using the well-established ELISA platform. In addition, autoantibodies to the zinc finger protein ZNF638 were previously identified in sera of patients with cutaneous T-cell lymphoma using the SEREX (serological identification of recombinantly expressed genes) approach [22]. A more recent study further confirmed our discovery of ZNF346 autoantibodies when sera of renal cancer patients were analysed [23]. Autoantibodies to other zinc finger proteins were also frequently detected in cancer [24] and autoimmune disease [25–27]; however, there is a paucity of literature on autoantibodies specific to zinc finger proteins ZNF700 and ZNF768. Since the presence of autoantibodies is not unique to colorectal cancer, further studies with other cancer types need to be conducted to show whether the panel of four ZNF proteins presented it this study is specific for colorectal cancer detection, or cancer detection in general.

Previous studies have reported a link between the presence of autoantibodies and patient survival. For example, autoantibody presence was associated with improved overall survival and prognosis in serous ovarian cancer, metastatic breast cancer and glioblastoma [28–30]. Furthermore, a study in pancreatic cancer reported a trend towards improved survival in patients with autoantibodies to the carboxy-terminal domain phosphatase CTDSP1 [31]. Correlations between cancer-specific autoantibodies and poorer prognosis have been reported in different types of cancers, including prostate cancer, malignant melanoma and cancer of the gingivo-buccal complex [32–35]. In the current study, we found no correlation between the presence of autoantibodies and patient survival. Similar findings have been reported in ovarian and breast cancer [36, 37] and may indicate that autoantibodies to zinc finger proteins are more suitable for diagnostic or screening purposes rather than disease prognosis.

In this study, we found that autoantibodies to individual zinc finger proteins were shared between several CRC patients. Since the ZNF proteins belong to one of the most common classes of human proteins and are defined by their evolutionary conserved zinc finger motifs [38], we set out to investigate *in silico* whether potential autoantibody epitopes can be found within their protein sequences. While autoantigens can present as discontinuous, conformational or linear epitopes [39], we focused our epitope prediction analyses on linear epitopes, as the protein arrays used for the discovery of ZNF346, ZNF638, ZNF700 and ZNF768 autoantibodies comprise of significantly denatured, highly unfolded polypeptides associated with optimal recognition of linear epitopes [17, 40]. Our analysis revealed an accumulation of linear B-cell epitopes shared between the classical C2H2 zinc finger motifs of ZNF700 and ZNF768. Only one linear B-cell epitope was predicted within the ‘matrin-like’ motif of ZNF346, and it was not shared with the ‘matrin-like’ zinc finger protein ZNF638. These results suggest an elevated immunogenic potential of the classical C2H2 zinc finger motif, which is also supported by other studies [24]. However, although the classical C2H2 zinc finger motif is the most abundant among all zinc finger protein classes [38], no further C2H2 motif zinc finger proteins were discovered as CRC-specific autoantigens among other 200 ZNF proteins deposited on the protein arrays, suggesting that the C2H2 motif may enhance the immunogenicity of the autoantigen without loosing the immunogenic uniqueness of the corresponding epitope.

A limitation of our study relates to the composition of the non-cancer control group. Although patients enrolled as control group in this study were selected against all key clinical determinants that may influence their autoantibody immunity, including absence of systemic inflammatory disease, autoimmune disease and immunosuppressive medication, as well as the absence of colorectal cancer and history of cancer, the control group showed a discrepancy in median age when compared to the CRC group. Although the prevalence of autoantibodies does not usually increase non-specifically as a result of aging, autoantibodies may be found more frequently in the elderly, as systemic inflammatory disease and autoimmunity becomes prevalent with age [41]. It is important to note that the original discovery of autoantibodies to ZNF346, ZNF638, ZNF700 and ZNF768 in our previous colorectal cancer study was performed in a sex and age matched case-control cohort with median age of 62 for CRC and 61 for controls, thereby excluding autoantibodies specific to age-related illnesses.

Our study demonstrates that recombinant human zinc finger proteins expressed in *E*. *coli* may serve as useful and convenient capture antigens for the detection of circulating autoantibodies in CRC. Cancer progression correlates with enhanced dysregulation of key signalling pathways and therefore different stages of cancer may trigger different autoantibody profiles. In this study however, we showed that ZNF-specific autoantibodies were found in cancer patients of all stages. Since early detection of cancer is a critical contributing factor for patient outcomes, a combination of markers encompassing all stages of the disease may aid in an earlier detection and improved patient survival. Another interesting feature of our study was the *in silico* analysis of B-cell epitopes associated with the zinc finger motifs. Knowledge of such epitopes may improve the quality of future autoantigen-based diagnostic assays, however it may require further *in vitro* epitope mapping studies.

In summary, we show a multi-marker ZNF autoantibody assay that provides a potential tool for improving cancer detection, which could be used for cancer screening as well as diagnosis, monitoring of cancer progression and therapeutic interventions.

## Supporting Information

S1 Table Zinc finger motif sequences for ZNF346, ZNF638, ZNF700 and ZNF768. (XLSX)

S2 Table Local similarity (SIM) based alignments between ZNF protein pairs. (XLSX)

S3 Table BepiPred linear epitope prediction. (XLSX)
